# Obituary

**Published:** 1853-07

**Authors:** 


*Obituary.*—Died, on the 18th ultimo, Dr. Joseph Peabody, aged 59 years.

Dr. Peabody established himself as a practitioner, in Buffalo, about eight years ago. He had previously, for many years, been engaged in practice in

West Norwich, Conn. During the few years of his residence in this city, he acquired many friends, by whom the loss occasioned by his decease will be painfully felt. Resolutions of sympathy were adopted at a special meeting of the Erie Co. Med. Society, and a large number of the members of the society testified their respect for the deceased by their attendance at the funeral solemnities, which were held at the Central Presbyterian church, on which occasion the Rev. Dr. Lord, pastor of the church, delivered an impressive discourse.

Dr. Peabody was for many years before his death a professing Christian, and was sustained in his last brief illness by his religious faith.

